# The Role of Diet and Weight Loss in Improving Secondary Hypogonadism in Men with Obesity with or without Type 2 Diabetes Mellitus

**DOI:** 10.3390/nu11122975

**Published:** 2019-12-05

**Authors:** Vito Angelo Giagulli, Marco Castellana, Isanna Murro, Carla Pelusi, Edoardo Guastamacchia, Vincenzo Triggiani, Giovanni De Pergola

**Affiliations:** 1 Section of Internal Medicine, Geriatrics, Endocrinology and Rare Disease, Interdisciplinary Department of Medicine, University of Bari, School of Medicine, Policlinico, 70124 Bari, Italy; 2 Outpatients Clinic of Endocrinology and Metabolic Disease, Conversano Hospital, 70014 Bari, Italy; 3 National Institute of Gastroenterology “S. De Bellis”, Castellana Grotte, 70013 Bari, Italy; 4 Clinical Nutrition Unit, Medical Oncology, Department of Biomedical Science and Human Oncology, University of Bari, School of Medicine, 70124 Bari, Italy; 5 Division of Endocrinology and Center for Applied Biomedical Research, Department of Medical and Surgical Sciences, Alma Mater Studiorum, University of Bologna, S. Orsola-Malpighi Hospital, 40138 Bologna, Italy

**Keywords:** obesity, hypogonadism, testosterone, diet, physical activity, insulin resistance, type 2 diabetes mellitus

## Abstract

Despite growing recognition of the issue, obesity represents one of the most common public health problems, and its rates are still increasing globally. Among the number of comorbidities and complications associated with obesity, hypogonadism is listed, and this disorder, although frequently neglected, is characterized by a relevant impact on both quality of life and life expectancy. It is generally accepted that hypogonadism secondary to obesity is functional since it is reversible following weight loss. This review summarizes all current research examining the bidirectional relationship between excess body weight and low testosterone levels. Specifically, it evaluates the role that diet, with or without physical activity, plays in improving body weight and hypogonadism in adult and elderly men with obesity, with or without type 2 diabetes mellitus.

## 1. Introduction

Obesity is one of the major public health issues globally, since it is associated with a higher morbidity [1] and mortality [2], leading to a relevant economic impact on the affected people, their families, and the health care system [3]. Subjects with obesity have a higher risk of a number of comorbidities, including insulin resistance, type 2 diabetes mellitus (T2DM), dyslipidemia, hypertension, cardiovascular disease, some types of cancer, hyperuricemia, deep vein thrombosis and pulmonary embolism, sleep-related breathing disorders, osteoarthritis, and hypovitaminosis D [4,5,6]. In males, obesity represents the clinical condition most strongly associated with hypogonadism, leading to erectile dysfunction and infertility due to oligospermia/azoospermia [7,8,9]. In addition, in a vicious cycle, male hypogonadism impairs body composition (reduced muscle mass, higher fat mass), fat metabolism, bone mineralization, and cognitive function. Particularly, central fat accumulation has been linked with all the disorders above, and waist circumference has been found as the most reliable predictor of both lower serum testosterone (T) and risk of developing metabolic and cardiovascular diseases [10,11,12]. The first-line strategy for the management of overweight and obesity is represented by lifestyle interventions, including diet and physical activity; in patients not achieving targets and meeting specific criteria, pharmacological therapy and bariatric surgery should be considered [6,13].

In the present narrative review, the pathophysiology of functional secondary hypogonadism is summarized, the impact of hypogonadism on body weight evaluated, and the role of lifestyle interventions with a special focus on diet in improving this disorder in men with obesity with or without type 2 diabetes mellitus discussed. Also, the possible effects of T supplementation in these patients is reported.

## 2. Pathophysiology of Functional Secondary Hypogonadism

In the last few years, a new classification of hypogonadism has been proposed. Together with the organ responsible for the disorder (either testis in primary forms or hypothalamus/pituitary gland in secondary forms), the mechanism underlying hypogonadism should be considered as functional and organic forms differentiated. To be classified as functional, hypogonadism should be reversible following the treatment of underlying causes. No structural, destructive, or congenital disease should affect the hypothalamic–pituitary–testicular axis (HPT) [14,15,16].

Among adult males, the most common form of hypogonadism is functional secondary hypogonadism, also known as dysmetabolic hypogonadotropic hypogonadism or male obesity secondary hypogonadism (MOSH). The pathogenesis of this disorder is multifactorial and generally related to chronic diseases, including obesity, T2DM, and the metabolic syndrome [15,16]. Regardless of the latter diseases, an inverse relationship between T and excess body weight has been reported in men of all ages, with lower T levels found in subjects with higher body mass index (BMI) [7]. Indeed, low total testosterone (TT) primarily reflects reduced concentrations of sex hormone-binding globulin (SHBG) in mild obesity, with no clear evidence of clinical androgen deficiency or defective spermatogenesis [17]. Conversely, more severe forms of obesity lead to overt hypogonadotropic hypogonadism, due to the suppression of the HPT axis, and lower levels of both TT and free testosterone (FT) and even of inhibin B [17,18,19,20,21,22,23]. Interestingly, this mechanism of HPT axis damage seems to be already present at puberty in boys with obesity [24]. Gonadotropins (follicle-stimulating hormone [FSH] and luteinizing hormone FSH [LH]) may be inappropriately normal or low in mild obesity [8], and a specific decrease in pulse amplitudes of LH has been reported in middle-aged men with severe obesity [25]. Several factors are involved in the pathogenesis of this form of hypogonadism, in particular, a role of estradiol, insulin, leptin, and other pro-inflammatory cytokines has been proposed. A brief summary of the contribution of each hormone is reported in the following sections.

### 2.1. Estradiol

In men with obesity, a part of androgens is converted to estrogens through peripheral aromatization in adipose tissue [26]. The most represented estrogen is estradiol, and this has been suggested to induce functional secondary hypogonadism due to the suppression of the HPT axis [27,28]. Indeed, early studies showed higher estradiol concentrations in young to middle-aged subjects with obesity compared to lean controls [29], and following studies reported the treatment of hypogonadal subjects with obesity with selective estrogen receptor modulators (SERM) or anti-aromatase inhibitors (AI) to be successful in stimulating the HPT axis and in achieving physiological T levels [30,31]. Based on these findings, it was hypothesized that low TT levels in subjects with obesity and hypogonadism are due to the estradiol-related HPT axis suppression and low levels of SHBG.

In recent years, this hypothesis has been questioned. Different studies found low levels of both estradiol and TT in men with obesity [32] as well as in hypogonadal men with T2DM [33]. Indeed, given that up to 40% of circulating estradiol is synthesized from the peripheral conversion of T to estradiol, it is much more likely that men with obesity and low serum T may have low estradiol levels too [25,34]. Accordingly, no changes in estradiol levels after weight loss were reported [18], thus supporting the picture of hypogonadal men with obesity as not being characterized by a chronic estrogen excess [35]. Given the findings above, one would raise the question whether a vicious cycle is there for obesity and estrogens, as the one described for T. Indeed, in preclinical studies, adipose tissue inflammation and insulin resistance were found to reduce aromatase expression, which returned to normal levels with improvements in the factors above [36]. In addition, adequate estradiol concentrations were linked with favorable metabolic effects, including reduced white adipose tissue accumulation, preserved insulin sensitivity in skeletal muscles and liver, reduced apoptosis of pancreatic β-cells driven by oxidative stress, amyloid polypeptide toxicity, or lipotoxicity, and enhance glucose-stimulated insulin biosynthesis. Furthermore, estrogens act on the central nervous system, particularly in the hypothalamus, leading to a decrease food intake [37].

In all, circulating estradiol is primarily dependent on T, and there is growing evidence that its levels tend to decrease rather than to increase along with the progressive increase of fat mass [35]. To date, conflicting data are reported in the literature on the contribution of estrogens to the pathogenesis of functional secondary hypogonadism and the possible mechanisms not definitely explained.

### 2.2. Insulin

In vitro studies showed that insulin stimulates the secretion of gonadotropin-releasing factors (GnRH) from the hypothalamus, gonadotropin from the pituitary gland, and T from the Leydig cells in the testis [38,39,40]. Obesity and T2DM are characterized by insulin resistance and, in those patients in which this is not offset by the hyperinsulinemia, hypogonadism can be found due to the lack of response of the HPT axis. It is worth noting that in patients with T2DM, a suppression of testicular function rather than the HPT axis is possible, and 5% of men with T2DM present with hypergonadotropic hypogonadism. However, the largest part is characterized by functional hypogonadal hypogonadism mainly due to concomitant obesity. Other mechanisms have also been proposed to describe the relationship between insulin and low T. Particularly, insulin resistance and hyperinsulinemia are known to inhibit hepatic SHBG secretion in vitro and in vivo [41]. This protein bounds 40–50% of total testosterone and plays a key role in the crosstalk between hypogonadism and metabolic disorders. Low levels of SHBG are commonly found in patients with obesity or type 2 diabetes mellitus. Since SHBG influences the bioavailability of testosterone, a reduction in SHBG is associated with transient higher FT which is aromatized to estradiol, possibly inducing negative feedback on the hypothalamic–pituitary–testicular axis [42]. Also, in patients with obesity, metabolic syndrome, diabetes mellitus, dyslipidemia, and hypertension, a common comorbidity is represented by non-alcoholic fatty liver disease (NAFLD), which includes non-alcoholic fatty liver (NAFL) and non-alcoholic steatohepatitis (NASH). This is a strong predictor of progression to type 2 diabetes mellitus and is an independent risk factor irrespective of age and BMI [43]. Given that testosterone undergoes hepatic metabolization and its levels are influenced by circulating SHBG which is secreted by the liver, it has been hypothesized that NAFLD may play a key role in secondary hypogonadism [44,45]. Accordingly, several observational studies and a meta-analysis demonstrated a significant correlation between being diagnosed with NAFLD and low levels of both TT and SHBG. There were not enough data to draw a definitive conclusion on FT [45,46]. However, a recent study showed that mechanisms linking inflammation to hypothalamic–pituitary downregulation and then to overt secondary hypogonadism described in animal models apply to humans too [47]. Eighty patients with obesity were included, and sex hormone levels and liver disease were evaluated by the means of gold standard techniques, represented by mass spectroscopy and liver biopsy, respectively. Compared to patients with NAFL, those with NASH showed lower serum FT levels and higher levels of free estradiol/FT and FT/LH ratios, indicating an overt condition of hypogonadism driven by an increase in adipose tissue aromatization. Thus, while confirming this relationship between low testosterone and NAFLD, this provided a new and interesting pathophysiogical mechanism. As a matter of fact, it demonstrated that the grade of inflammation, as well as the severity of liver disease, influence androgen levels [48]. It is worth noting that, to date, there are not enough data in order to support a bidirectional correlation between hypogonadism and NAFLD [45].

### 2.3. Leptin

An important role in the pathogenesis of functional secondary hypogonadism is played by the visceral adipose tissue, in which a variety of circulating mediators (including leptin and pro-cytokines) contributing to the suppression of the HPT axis at multiple levels is synthesized [17,41,49,50]. Leptin is a major determinant of energy homeostasis and key in the regulation of male fertility. Concerning to former aspect, it acts both on the central nervous system, controlling food intake, energy expenditure, and fat distribution, and peripherally, favoring insulin sensitivity, lipolysis, and free fatty acids (FFA) oxidation [51]. Concerning the latter aspect, it acts on the HPT axis at multiple levels. In the hypothalamus, it stimulates the release of kisspeptin from forebrain neurons, which induces GnRH and gonadotropin secretion [52,53]. In the testis, it inhibits basal and hCG-stimulated T production in adult rat testis [54,55]. These mechanisms seem to be in conflict with each other; furthermore, considering the former mechanism, hypogonadism would be hard to explain, given that that the adipose tissue is the main source of leptin and subjects with obesity are characterized by high levels of leptin. However, as for insulin and T2DM, the main link between leptin and hypogonadism could be represented by leptin resistance, leading to either defects in the intracellular signaling of the leptin receptor or an abnormal leptin transport across the blood–brain barrier [56]. In male obese hypogonadal animals, leptin resistance was shown to reduce GnRH secretion through a downregulation of kisspeptin gene expression and kisspeptin receptors [57]. On the contrary, it is believed that leptin sensitivity is maintained in the testis, thus inhibiting steroidogenesis [54]. In conclusion, in male patients with obesity, the combination of leptin resistance in the hypothalamus and a preserved leptin sensitivity in the testis leads to hypogonadism [58].

### 2.4. Other Pro-Inflammatory Cytokines

As already stated, obesity and metabolic syndrome are characterized by chronic inflammation and high levels of cytokines that are known to impact on Leydig cell steroidogenesis and T levels either directly interfering with the HPT axis or indirectly altering the insulin transduction pathway [59,60,61]. Besides leptin, these include tumor necrosis factor-alpha (TNF-α), interleukin-1β (IL-1β), interleukin-6 (IL-6), and interleukin-8 (IL-8). In vitro studies showed TNF-α and IL-6 to be able to suppress GnRH and LH secretion [62,63]. Also, in the testis, TNF-α, IL-1β, and IL-6 were associated with a dose-dependent decline in steroidogenesis in TM3 Leydig cells [64]. Furthermore, in rat models of diet-induced obesity, high levels of TNF-α, IL-1β, and IL-6 were associated with the disruption of testicular morphology, with a reduction in seminiferous tubule diameter, epithelial and germ cell atrophy and apoptosis, abnormal adhesions between Sertoli and spermatogenic cells and the blood–testis barrier disassembly [65]. On the other hand, even if IL-8 exerts a stimulating effect on TM3 Leydig cell growth, it appears to minimally affect T production and blood concentrations [64]. Recently, other cytokines, such as resistin and visfatin, have been assessed for their possible involvement in the alteration of the HPT axis through inducing an insulin resistance state [66].

The clinical relevance of targeting cytokines for the management of functional secondary hypogonadism was recently shown in a randomized controlled trial (RCT) conducted in hypogonadal men with obesity: the administration of an anti-inflammatory treatment with anakinra (recombinant human IL-1 receptor antagonist) led to an increase in endogenous T levels [67]. Overall, these data support the connection among chronic inflammation, high levels of cytokines, and hypogonadism, even if the exact mechanisms and site of action on the HPT axis are still a matter of debate.

## 3. Preclinical Studies On the Impact of Hypogonadism on Different Tissues

Animal models are useful in evaluating the impact of a specific molecule on a specific tissue. To achieve this target, an interesting option is represented by the generation of knockout models. Particularly, in the setting of hypogonadism, this result can be achieved through the knockout of the androgen receptor (AR). Preclinical studies linked sex hormones (especially T) with glucose homeostasis and insulin sensitivity. Indeed, endocrine pancreas (α-cells and β-cells), liver, muscle, adipose tissue, and neuronal tissue express the AR and the estrogen receptor (ER). Despite both α-cell and β-cell presenting AR, the effects of androgens in α-cell is still unknown. Conversely, the selective knockout of the AR in mice β-cells was associated with glucose intolerance due to a reduction in glucose-stimulated insulin secretion, leading to hypoinsulinemia, glucose intolerance, and hyperglycemia. Moreover, in these models, T treatment could not improve glucose-stimulated insulin secretion [68]. The selective knockout of the AR in mice liver was associated with steatosis, insulin resistance, increased lipogenesis, and reduction in FFA oxidation [69]. The male genomic AR knockout mice were associated with reduced muscle mass, strength, and fatigue resistance [70]. The same animal model was characterized by visceral obesity and both insulin and leptin resistance at an advanced age. Of note, AR and ER density varies according to the type of adipose tissue more than sex, and visceral adipose tissue presents with higher AR density than subcutaneous adipose tissue, highlighting the crucial role of T in modulating body composition and metabolism [71]. Then, several selective adipose tissue-specific AR knockout models were generated. A first model was generated using adipocyte-specific fatty acid binding 4 protein (aP2) Cre mice and provided a phenotype of hyperleptinemia without leptin resistance [72]. Another model was generated by crossing the same aP2-Cre with a different floxAR line, and it was characterized by hyperinsulinemia in the absence of obesity. However, these mice presented increased susceptibility to visceral obesity, hyperglycemia, and impaired insulin secretion whenever they started a high-fat diet [73]. Finally, a selective knockout of the AR in the mice central nervous system was generated using synapsin I Cre and showed reduced insulin sensitivity, increased visceral obesity, serum triglycerides, and FFA [74]. Overall, the global AR knockout models suggest that the AR regulates a number of pathways, and this regulation might occur through AR actions in organs other than fat tissue, including the pancreas, muscle, and the central nervous system.

## 4. Lifestyle Interventions for the Management of Functional Secondary Hypogonadism

Although obesity is responsible of functional hypogonadotropic hypogonadism, a decrease of serum T levels is predictive of metabolic syndrome, unfavorable changes of body composition (increase of fat mass and decrease of fat free mass), lower insulin sensitivity, higher waist circumference, fasting glucose, triglyceride, and LDL-cholesterol levels at all ages [75]. Accordingly, low SHBG, T, and other sex steroids in community-dwelling middle-aged to elderly men and androgen deprivation therapy, as in the case of prostate cancer treatment, were associated with a higher incidence of T2DM and cardiovascular diseases [76,77,78]. On the other hand, testosterone replacement therapy (TRT) was associated with reduced fat mass and increased lean mass in hypogonadal dysmetabolic patients [79]. In fact, T plays a critical role in the regulation of energy utilization, including adipogenesis, carbohydrate and fat metabolism, and nitrogen retention [80].

Weight loss, however obtained (by diet, physical exercise, pharmacological therapy, or bariatric surgery), reverses functional hypogonadotropic hypogonadism secondary to obesity to eugonadism and induces an increase in both TT and FT [17,22,81]. In particular, at least 10% of weight loss is required to achieve a significant increase in circulating T in most men with obesity and in improving clinical features related to androgen deficiency [17]. In general terms, lifestyle measures are less effective in inducing weight loss and are associated with less evident increases in circulating T [17]. However, any second-line intervention for obesity (either pharmacological therapy or bariatric surgery) should always be considered as an add-on to lifestyle measures, including diet and physical activity. In addition, one longitudinal study performed in men with obesity submitted to bariatric surgery has shown that the degree of weight loss was more closely associated with improvements in sexual function rather than with the increase in T levels [82].

### 4.1. Effects of Lifestyle: Diet Only

An increase in serum androgens levels was reported following weight loss achieved through very-low-calorie diets (VLCD) or low-calorie diets (LCD). Stanik et al. investigated the hormonal profiles after a very restricted dietary protocol (supplemented fasting program with 320 kcal/day) for 8–20 weeks in 24 moderately men with obesity, and they observed that a mean weight loss of 19 kg induced the normalization of TT and FT levels [83]. Another study evaluated 38 men with obesity divided into two groups: 19 subjects were treated for 12 weeks with a VLCD, with total energy intake of approximately 800 kcal/day, and subsequently for 10 weeks with a behavioral variation; the remaining 19 subjects were untreated and were considered as control subjects. The rapid weight loss induced by VLCD resulted in an increase in SHBG and TT levels, and a decrease in serum insulin and leptin levels; all these changes were maintained until the end of the follow-up period (8 months). Interestingly, when the changes induced by maintained weight loss were evaluated in a backward regression analysis, the decrease in insulin resistance was the most significantly parameter related to serum T levels [84]. These data are in line with the results of another study, evaluating serum sex hormones levels, sexual function, and low urinary tract symptoms (LUTS) assessed through IPSS score before and after 8 weeks in 51 men with visceral obesity with or without T2DM assigned to LCD (850–900 kcal/day) compared to a control group of 25 men without T2DM but with similar BMI and waist circumference assigned to their usual diet. Weight loss was significantly correlated with increased insulin sensitivity, TT, sexual function and sexual desire item scores; moreover, the two latter endpoints were directly associated with the level of waist circumference reduction [85]. The effects of the weight loss induced by diet on hormonal, sexual, and urinary functions were also confirmed in a cohort of 31 men affected by obesity and T2DM, showing improvements in all endpoints either in an initial 8-week LCD or in the following 44-week maintenance high protein, low-fat, and reduced-carbohydrate diet having a decrease in daily intake of approximately 600 kcal/day. This study also showed a significant decrease in inflammatory markers, such as C-reactive protein and IL-6, and an improvement in endothelial function evaluated through brachial artery flow-mediated dilatation and soluble E-selectin levels. Thus, mechanisms other than higher T are possibly involved in the improvements in sexual and urinary functions in men with obesity [86]. Finally, Niskanen et al. experimented with the effect of rapid weight loss obtained by a 9-week VLCD followed by a 12-month weight maintenance in a cohort of 58 men with visceral obesity. An increase in serum SHBG levels during VLCD and a little decrease during weight maintenance, staying however higher than baseline, were found; FT was significantly increased, both following the rapid weight loss induced by VLCD and at the end of the 1-year weight maintenance period, whereas TT showed intermediate variations between those of SHBG and FT [87]. In obese men, a rise in SHBG (and therefore TT) is well known after weight loss as a result of insulin sensitization. FT or bioavailable testosterone measurement is needed for the appropriate assessment of gonadal status. Relevant data on the impact of diet on FT have been published in recent years. A first meta-analysis found a low-calorie diet to be associated with an increase in TT, SHBG, and FT. It is worth noting that in this meta-analysis, the results on the latter outcome were based on only two studies evaluating diet as intervention [81,84,87]. The results were updated in a following meta-analysis published in 2019 by the same group. In this study, the results of eight studies were pooled, and the significant increase in FT was confirmed [88]. In all, a limited number of studies are reported in the literature on the impact of diet on FT. Based on available data, a positive correlation should be expected between weight loss and change in FT. These data strongly emphasize that diet is key as the first-line treatment for functional hypogonadal hypogonadism secondary to obesity.

Since cholesterol is a precursor of T, an interesting topic is the possible effects of vegetarian diets on the production of T. Surprisingly, very few not-recent studies investigated this aspect and conflicting findings were reported. One study found lower levels of T and estradiol in vegetarian men compared to omnivores men [89]. Other studies showed similar concentrations of TT, FT, and estradiol in the same populations, despite a higher SHBG in the former [90,91]. In all, apart from the clear higher SHBG levels in vegetarians, which are mainly due to high fiber intake, clear evidence on the effects of vegetarian diets on T is currently lacking. Western-style diets (WSDs) are characterized by a high intake of cholesterol and protein; thus, it represents an interesting model to examine whether food habits may change T levels in men. It is worth noting that WSD is highly heterogeneous in quantities and qualities of fat (saturated vs. unsaturated), carbohydrates (high glycemic vs. low glycemic), and protein content, and recent evidence suggests that imbalance in the levels of sex steroids is associated with WSD. However, whether this alteration is mediated through obesity and insulin resistance at least in part, there is no clear demonstration that WSD per se is responsible for lower T levels and infertility [92].

### 4.2. Effects of Lifestyle: Diet and Physical Activity

Several studies reported that physical activity is associated with an increased endogenous T in men with obesity. Kumagai et al. evaluated the impact of aerobic exercise on T levels in 28 men with overweight/obesity. After 12 weeks, an increase in TT and FT in the latter group was found, with vigorous exercise independently associated with improvements in TT [93]. Given the results of diet and physical activity when assessed as single interventions, a number of studies were performed to assess their combined effect. Armamento-Villareal et al. evaluated the impact of lifestyle interventions on hormone levels in frail older men with obesity. After 12 months, weight loss was not associated with improvements in either TT or FT levels. However, the clinical relevance of these findings could be limited, given the inclusion criteria of the study population. Indeed, in frail and older men with obesity, weight loss is not the best approach for raising T levels because it can worsen age-related muscle and bone loss [94]. Recently, De Lorenzo et al. evaluated the effects of a 10% weight loss achieved through diet and physical activity on the hormonal profile of MOSH patients with total testosterone below 12.1 nmol/L (349 ng/dL). The prescribed diet consisted of a “Mediterranean” style diet, with all macronutrients present in each of the five meals. This was characterized by high consumption of fresh vegetables, fruits, and extra virgin olive oil, and proteins were given through legumes, cereals, and fish [95]. Overall, it was a hypocaloric (170–250 kcal/day reduction compared to basal metabolism), high-protein (1.5 g/day of ideal body weight) diet; calories were given through carbohydrates (45–50%), proteins (20–25%), and fat (30%; saturated fat <7%, polyunsaturated fatty acids 10–20%, monounsaturated fatty acids 10–20%, cholesterol <300 mg/day). Fiber intake was 25–30 g/day, sodium intake was <5 g/day; no alcoholic beverages were allowed. Concerning physical activity, subjects were prescribed with 150 min/week of mild intensity aerobic (50–70% of max heart rate) activity and/or 90 min/week of high-intensity (>70% max heart rate) activity to be practiced at least three days/week. After 3 ± 1 months, a significant increase in TT was observed, together with a reduction in fat mass percentage [96]. The largest study examining the effects of weight changes on T concentrations was the Diabetes Prevention Program (DPP) trial. Eight-hundred-eighty-six adult men with a mean BMI 32 kg/m^2^ and a mean age 52 years were randomized to placebo twice daily (*n* = 278), 850 mg metformin twice daily (*n* = 315), or intensive lifestyle intervention (*n* = 293). After 12 months, the weight loss was −0.12 kg, −2.73 kg, and −7.87 kg, respectively. TT increased only in the latter group by 1.15 nmol/L [97]. In synthesis, this demonstrates that lifestyle interventions are effective in improving body composition, hormonal and metabolic profiles. Therefore, they should represent the first choice in the management of obesity and functional secondary hypogonadism.

Concerning the effects of lifestyle measures on semen quality, there is still very little information. One study evaluated 43 men with obesity before and after 14 weeks of a weight loss program based on a healthy diet and physical exercise. Weight loss was significantly associated with an increase in total sperm count, semen volume, TT, and serum SHBG levels, with those men achieving the largest weight loss showing normal sperm morphology [98].

### 4.3. Effects of Adding Testosterone Replacement Therapy to Lifestyle Interventions

As already stated, lifestyle interventions should be regarded as relevant tools for the management of functional secondary hypogonadism. However, the question arises as to whether TRT can enhance the benefits of lifestyle changes and prevent the diet-associated loss of muscle mass. One RCT performed by Hidreth et al. in healthy older men with low-normal T levels (200–350 ng/dL) showed that T supplementation compared to placebo improved body composition in all patients; however, no effect on functional performance or strength in those patients allocated to progressive resistance training was found, while an improved upper body strength only in those patients allocated to no exercise was reported [99]. In another RCT, no additional benefit on body weight, BMI, and waist circumference was found when T treatment or placebo were given to men with obesity and obstructive sleep apnea on VLCD. It is worth noting that the baseline T levels in each group were 13.2 and 13.4 nmol/L, respectively; whether these results could be considered applicable to all patients with functional secondary hypogonadism is unclear [100]. In a recent 56-week RCT performed by Ng Tang Fui et al., middle-aged (median age 53 years) men with obesity and low TT (mean 6.9 nmol/L) were subjected to a rigorous 10-week VLCD phase followed by 46 weeks of weight maintenance and were randomized at baseline to TRT or placebo. Both groups lost a similar amount of body weight at the end of the VLCD phase. However, men allocated to T had greater reductions in fat mass (between-group difference −2.9 kg) and visceral fat (−2678 mm^2^), and preservation of lean mass (+3.4 kg), compared to placebo. Thus, TRT prevented the diet-associated loss in lean mass without increasing the diet-associated weight loss [101]. When the same patients were evaluated more than one year after the RCT completion, in those patients in which TRT was withdrawn, T concentrations fell back to baseline concentrations. Two-thirds of the weight loss was regained, the effect of TRT on body composition was not apparent anymore, and differences in fat and lean mass between groups were no longer maintained [102]. This suggests that relatively short-term (56 weeks) TRT is not associated with sustained benefits and that long-term therapy should be considered to maintain the achieved results [16,101,102]. However, TRT can be considered to overcome fatigue and engage a healthy lifestyle in some groups of patients, in line with results of other studies [103].

As far as the mechanisms involved in the effects of T on changes in body composition is concerned, two options can be considered: the first is represented by its direct effects on adipose and skeletal muscle cells; the second may well be the spontaneous increase of physical activity observed in men on TRT [29,101]. This treatment was associated with lower leptin levels, suggesting a decrease in adipose tissue mass and/or a lessening of leptin resistance [22]. On the other hand, it is to note that TRT does not significantly influence circulating concentrations of ghrelin, glucagon-like peptide-1, gastric inhibitory polypeptide, peptide YY, pancreatic polypeptide, and amylin, suggesting that its effects on body composition are unlikely to be mediated by changes in modulators of appetite and energy homeostasis [29]. Regarding sexual function, TRT improves this aspect independently of the effect on weight loss [104]. In the large T trials, sexual function improved modestly with TRT, suggesting that even older men with a relatively high comorbid burden (T2DM or previous myocardial infarction) can respond to T [105].

Hypogonadism is associated with impaired physical activity. A recent systematic review found TRT to be associated with increased physical function without any impact on muscle strength [106]. These findings are not in line with one reported by Storer et al. in which three years of TRT was associated with an increase in stair-climbing power, muscle mass, and power [107]. Further studies to address this issue are needed.

Lower TT and FT are generally found in patients with T2DM [108,109]. However, the presence of this disorder does not affect the positive effects of TRT. A prospective study, performed for over five years in 181 men with obesity, including 72 patients affected by T2DM, treated by parenteral testosterone undecanoate 1000 mg/12 weeks, showed a significant and sustained reduction in body weight, BMI and waist circumference. In addition, this study showed that restoring serum testosterone to normal in hypogonadal men with obesity is beneficial with regards to the metabolic syndrome: fasting glucose declined over the first 18 months and then stabilized, with similar trend for serum total cholesterol, LDL cholesterol, and triglycerides; HDL increased slightly but significantly; diastolic and systolic blood pressure decreased. This pattern was similar in patients with or without T2DM and is in line with earlier studies [110,111].

Although male hypogonadism can be considered an independent risk factor for the development of metabolic syndrome, cardiovascular disease, and all-cause mortality, TRT presents some concerns, especially whenever it is taken into consideration for the treatment of elderly men suffering from overt hypogonadism [112,113,114]. Indeed, elderly men can be affected by different metabolic disorders, which favors the appearance of clinically important cardiovascular events, causing a state of fragility and vulnerability [115]. However, several clinical studies and a recent meta-analysis of randomized controlled studies proved that TRT improved waist circumference, body composition, some metabolic parameters such as homeostatic model assessment (HOMA) and glycemic control, and reduced the progression from prediabetes to overt diabetes mellitus type 2 in adult dysmetabolic men [116,117,118]. In addition, a meta-analysis of pharmaco-epidemiological studies demonstrated that patients treated with T compared with controls showed a reduction of both mortality and mobility, especially due to acute myocardial infarction, stroke, acute coronary syndromes, and heart failure. Conversely, data from randomized controlled studies suggest that TRT is not able to reduce cardiovascular risk, however, when the therapy with T is correctly applied, at least it is not associated with an increased cardiovascular risk. All in all, there is a need for adequately powered placebo-controlled studies with cardiovascular disease as the primary endpoint carried out in men with late-onset hypogonadism in order to strengthen the conclusion that TRT is not associated with higher cardiovascular risk [119]. Another delicate issue when TRT is considered is represented by prostatic cancer. Cunningham et al. reported that, in older men with symptomatic hypogonadism, normalizing T levels for one year was associated with a small but statistically significant greater increase in Prostate-Specific Antigen (PSA) levels compared to placebo [120]. This may lead to subsequent urological consultation, prostate biopsy, and more frequent detection of prostate cancers, often represented by indolent lesions with consequent overtreatment and possible complications. It is worth noting that nearly half of the PSA elevations observed in this study were not confirmed when the analysis was repeated; furthermore, when patients with a confirmed elevation of PSA were referred to a urologist and prescribed with PSA repetition, an additional 50% of PSA elevations were not confirmed anymore, suggesting that PSA elevations are often the result of laboratory variability. Also, out of 790 patients during the 12 months of treatment and the six months of follow-up, only four prostate cancers were found, three of which in the testosterone replacement therapy arm and one in the placebo. In any case, the potential benefits and related risks of TRT should always be discussed with the patient, and elevated PSA values should always be confirmed by repeating the test [121,122,123,124]. In line with this, the Endocrine Society Guidelines for T treatment of hypogonadal men recommend a monitoring plan aiming at minimize the risk of unnecessary prostate biopsies avoiding the detection of indolent small tumors and leading at the same time to the identification of men who are at increased risk of having aggressive, potentially lethal prostate cancer. Urologic referral, in particular, is recommended for men who, in the first year of T treatment, have a confirmed increase in PSA values of 1.4 ng/mL or a confirmed absolute value of 4.0 ng/mL (or 3 ng/mL in men at high risk of prostate cancer) [16].

## 5. Conclusions

In patients with obesity and functional hypogonadotropic hypogonadism, weight loss has been shown to be effective in restoring eugonadism. Available studies emphasize the key role of lifestyle interventions as the first-line treatment to achieve improvements in body weight and secondary hypogonadism. Among diets, hypocaloric and high-protein diets with Mediterranean style seem to have the most favorable effect on T levels, T/estradiol ratio, sexual function, and sperm quality. Also, physical activity should always be recommended, given the positive influence on androgenicity. Lastly, TRT can be considered in specific groups of patients, in the knowledge that there is the lack of evidence of sustained benefits following its withdrawal. Further studies comparing different diet protocols and with a longer follow-up are needed. In particular, it would be of interest to focus on dietary protocols introduced in recent years, including the very-low-calorie ketogenic diet, the Paleolithic diet, and intermittent fasting for which we were not able to find a high level of evidence in the literature. The same holds true for physical activity, for which the definition of a dose-response correlation between duration or type of exercise and effects on T would be relevant.

## References

[1] Astrup A., Rossner S., Finer N., Van Gaal L. (2013). Obesity in Europe—Does anybody care?. Expert Opin. Pharmacother..

[2] Flegal K.M., Kit B.K., Orpana H., Graubard B.I. (2013). Association of all-cause mortality with overweight and obesity using standard body mass index categories: A systematic review and meta-analysis. JAMA.

[3] von Lengerke T., Krauth C. (2011). Economic costs of adult obesity: A review of recent European studies with a focus on subgroup-specific costs. Maturitas.

[4] De Pergola G., Silvestris F. (2013). Obesity as a major risk factor for cancer. J. Obes..

[5] Resta O., Foschino-Barbaro M.P., Legari G., Talamo S., Bonfitto P., Palumbo A., Minenna A., Giorgino R., De Pergola G. (2001). Sleep-related breathing disorders, loud snoring and excessive daytime sleepiness in obese subjects. Int. J. Obes. Relat. Metab. Disord..

[6] Garvey W.T., Mechanick J.I., Brett E.M., Garber A.J., Hurley D.L., Jastreboff A.M., Nadolsky K., Pessah-Pollack R., Plodkowski R. (2016). Reviewers of the AACE/ACE obesity clinical practice guidelines. American Association of Clinical Endocrinologists and American College of Endocrinology comprehensive clinical practice guidelines for medical care of patients with obesity. Endocr. Pract..

[7] Chambers T.J., Richard R.A. (2015). The impact of obesity on male fertility. Hormones.

[8] Davidson L.M., Millar K., Jones C., Fatum M., Coward K. (2015). Deleterious effects of obesity upon the hormonal and molecular mechanisms controlling spermatogenesis and male fertility. Hum. Fertil..

[9] Bieniek J.M., Kashanian J.A., Deibert C.M., Grober E.D., Lo K.C., Brannigan R.E., Sandlow J.I., Jarvi K.A. (2016). Influence of increasing body mass index on semen and reproductive hormonal parameters in a multi-institutional cohort of subfertile men. Fertil. Steril..

[10] Svartberg J., von Muhlen D., Sundsfjord J., Jorde R. (2004). Waist circumference and testosterone levels in community dwelling men. The Tromso study. Eur. J. Epidemiol..

[11] Balkau B., Deanfield J.E., Dépres J.P., Bassand J.P., Fox K.A., Smith S.C., Barter P., Tan C.E., Van Gaal L., Wittchen H.U. (2007). International day for the evaluation of abdominal obesity (IDEA): A study of waist circumference, cardiovascular disease, and diabetes mellitus in 168,000 primary care patients in 63 countries. Circulation.

[12] Lopez D.S., Qiu X., Advani S., Tsilidis K.K., Khera M., Kim J., Morgentaler A., Wang R., Canfield S. (2018). Double trouble: Co-occurrence of testosterone deficiency and body fatness associated with all-cause mortality in US men. Clin. Endocrinol..

[13] Durrer Schutz D., Busetto L., Dicker D., Farpour-Lambert N., Pryke R., Toplak H., Widmer D., Yumuk V., Schutz Y. (2019). European practical and patient-centred guidelines for adult obesity management in primary care. Obes. Facts.

[14] Tajar A., Forti G., O’Neill T.W., Lee D.M., Silman A.J., Finn J.D., Bartfai G., Boonen S., Casanueva F.F., Giwercman A. (2010). Characteristics of secondary, primary, and compensated hypogonadism in aging men: Evidence from the European Male Ageing Study. J. Clin. Endocrinol. Metab..

[15] Grossmann M., Matsumoto A.M. (2017). A perspective on middle-aged and older men with functional hypogonadism: Focus on holistic management. J. Clin. Endocrinol. Metab..

[16] Bhasin S., Brito J.P., Cunningham G.R., Hayes F.J., Hodis H.N., Matsumoto A.M., Snyder P.J., Swerdloff R.S., Wu F.C., Yialamas M.A. (2018). Testosterone therapy in men with hypogonadism: An Endocrine Society clinical practice guideline. J. Clin. Endocrinol. Metab..

[17] Grossmann M. (2018). Hypogonadism and male obesity: Focus on unresolved questions. Clin. Endocrinol. (Oxf.).

[18] Wu F.C., Tajar A., Pye S.R., Silman A.J., Finn J.D., O’Neill T.W., Bartfai G., Casanueva F., Forti G., Giwercman A. (2008). Hypothalamic-pituitary-testicular axis disruptions in older men are differentially linked to age and modifiable and modifiable risk factors: The European Male Aging Study. J. Clin. Endocrinol. Metab..

[19] Haring R., Volzke H., Felix S.B., Schipf S., Dorr M., Rosskopf D., Nauck M., Schofl C., Wallaschetofsky H. (2009). Prediction of metabolic syndrome by low serum testosterone levels in men: Results from the study of health in Pomerania. Diabetes.

[20] Zitzmann M. (2009). Testosterone deficiency, insulin resistance and the metabolic syndrome. Nat. Rev. Endocrinol..

[21] Gates M.A., Mekary R.A., Chiu G.R., Ding E.L., Wittert G.A., Araujo A.B. (2013). Sex steroid hormone levels and body composition in men. J. Clin. Endocrinol. Metab..

[22] Camacho E.M., Huhtaniemi I.T., O’Neill T.W., Finn J.D., Pye S.R., Lee D.M., Tajar A., Bartfai G., Boonen S., Casanueca F.F. (2013). Age-associated changes in hypothalamic-pituitary-testicular function in middle-aged and older men are modified by weight change and lifestyle factors: Longitudinal results from the European Male Ageing Study. Eur. J. Epidemiol..

[23] Saboor Aftab S.A., Kumar S., Barber T.M. (2013). The role of obesity and type 2 diabetes mellitus in the development of male obesity-associated secondary hypogonadism. Clin. Endocrinol..

[24] Nokoff N., Thurston J., Hilkin A., Pyle L., Zeitler P.S., Nadeau K.J., Santoro N., Kelsey M.M. (2019). Sex Differences in Effects of Obesity on Reproductive Hormones and Glucose Metabolism in Early Puberty. J. Clin. Endocrinol. Metab..

[25] Giagulli V.A., Kaufman J.M., Vermeulen A. (1994). Pathogenesis of the decreased androgen levels in obese men. J. Clin. Endocrinol. Metab..

[26] Bray G.A. (1997). Obesity and reproduction. Hum. Reprod..

[27] Baker H.W. (1998). Reproductive effects of nontesticular illness. Endocrinol. Metab. Clin. N. Am..

[28] T’Sjoen G.G., Giagulli V.A., Delva H., Crabbe P., De Bacquer D., Kaufman J.M. (2005). Comparative assessment in young and elderly men of the gonadotropin response to aromatase inhibition. J. Clin. Endocrinol. Metab..

[29] Schneider G., Kirschner M.A., Berkowitz R., Ertel N.H. (1979). Increased estrogen production in obese men. J. Clin. Endocrinol. Metab..

[30] Kovac J.R. (2016). Reproductive endocrinology: Oral enclomiphene citrate in obese men with hypogonadism. Nat. Rev. Urol..

[31] Loves S., Ruinemans-Koerts J., de Boer H. (2008). Letrozole once a week normalizes serum testosterone in obesity-related male hypogonadism. Eur. J. Endocrinol..

[32] Feldman H.A., Longcope C., Derby C.A., Johannes C.B., Araujo A.B., Coviello A.D., Bremner W.J., McKinlay J.B. (2002). Age trends in the level of serum testosterone and other hormones in middle-aged men: Longitudinal results from the Massachusetts male aging study. J. Clin. Endocrinol. Metab..

[33] Dhindsa S., Furlanetto R., Vora M., Ghanim H., Chaudhuri A., Dandona P. (2011). Low estradiol concentrations in men with subnormal testosterone concentrations and type 2 diabetes. Diabetes Care.

[34] MacDonald P.C., Madden J.D., Brenner P.F., Wilson J.D., Siiteri P.K. (1979). Origin of estrogen in normal men and in women with testicular feminization. J. Clin. Endocrinol. Metab..

[35] Wu A., Shi Z., Martin S., Vincent A., Heilbronn L., Wittert G. (2018). Age-related changes in estradiol and longitudinal associations with fat mass in men. PLoS ONE.

[36] Swerdloff R.S., Dudley R.E., Page S.T., Wang C., Salameh W.A. (2017). Dihydrotestosterone: Biochemistry, physiology, and clinical implications of elevated blood levels. Endocr. Rev..

[37] Gambineri A., Pelusi C. (2019). Sex hormones, obesity and type 2 diabetes: Is there a link?. Endocr. Connect..

[38] Burcelin R., Thorens B., Glauser M., Gaillard R.C., Pralong F.P. (2003). Gonadotropin-releasing hormone secretion from hypothalamic neurons: Stimulation by insulin and potentiation by leptin. Endocrinology.

[39] Pitteloud N., Hardin M., Dwyer A.A., Valassi E., Yialamas M., Elahi D., Hayes F.J. (2005). Increasing insulin resistance is associated with a decrease in Leydig cell testosterone secretion in men. J. Clin. Endocrinol. Metab..

[40] Salvi R., Castillo E., Voirol M.J., Glauser M., Rey J.P., Gaillard R.C., Vollenweider P., Pralong F.P. (2006). Gonadotropin-releasing hormone-expressing neurons immortalized conditionally are activated by insulin: Implication of the mitogen-activated protein kinase pathway. Endocrinology.

[41] Dhindsa S., Ghanim H., Batra M., Dandona P. (2018). Hypogonadotropic hypogonadism in men with diabesity. Diabetes Care.

[42] Pivonello R., Menafra D., Riccio E., Garifalos F., Mazzella M., de Angelis C., Colao A. (2019). Metabolic disorders and male hypogonadotropic hypogonadism. Front. Endocrinol. (Lausanne).

[43] Bellentani S., Scaglioni F., Marino M., Bedogni G. (2010). Epidemiology of non-alcoholic fatty liver disease. Dig. Dis..

[44] Giagulli V.A., Verdonck L., Deslypere J.P., Giorgino R., Vermeulen A. (1993). Comparison of plasma androgen glucuronide levels after percutaneous or peroral androgen treatment in men: Evidence for important splanchnic contribution to plasma 17 beta-hydroxyandrogen glucuronides. J. Clin. Endocrinol. Metab..

[45] Grossmann M., Wierman M.E., Angus P., Handelsman D.J. (2019). Reproductive endocrinology of nonalcoholic fatty liver disease. Endocr. Rev..

[46] Jaruvongvanich V., Sanguankeo A., Riangwiwat T., Upala S. (2017). Testosterone, sex hormone-binding globulin and nonalcoholic fatty liver disease: A systematic review and meta-analysis. Ann. Hepatol..

[47] Sarchielli E., Comeglio P., Squecco R., Ballerini L., Mello T., Guarnieri G., Idrizaj E., Mazzanti B., Vignozzi L., Gallina P. (2017). Tumor Necrosis Factor-α impairs kisspeptin signaling in human gonadotropin-releasing hormone primary neurons. J. Clin. Endocrinol. Metab..

[48] Van de Velde F., Bekaert M., Hoorens A., Geerts A., T’Sjoen G., Fiers T., Kaufman J.M., Van Nieuwenhove Y., Lapauw B. (2019). Histologically proven hepatic steatosis associates with lower testosterone levels in men with obesity. Asian J. Androl..

[49] Gianatti E.J., Grossmann M. (2019). Testosterone deficiency in men with type 2 diabetes: Pathophysiology and treatment. Diabet. Med..

[50] Rastrelli G., Filippi S., Sforza A., Maggi M., Corona G. (2018). Metabolic syndrome in male hypogonadism. Front. Horm. Res..

[51] Kelesidis T., Kelesidis I., Chou S., Mantzoros C.S. (2010). Narrative review: The role of leptin in human physiology: Emerging clinical applications. Ann. Intern. Med..

[52] Quennell J.H., Mulligan A.C., Tups A., Liu X., Phipps S.J., Kemp C.J., Herbison A.E., Grattan D.R., Anderson G.M. (2009). Leptin indirectly regulates gonadotropin-releasing hormone neuronal function. Endocrinology.

[53] George J.T., Veldhuis J.D., Tena-Sempere M., Millar R.P., Anderson R.A. (2013). Exploring the pathophysiology of hypogonadism in men with type 2 diabetes: Kisspeptin-10 stimulates serum testosterone and LH secretion in men with type 2 diabetes and mild biochemical hypogonadism. Clin. Endocrinol. (Oxf.).

[54] Isidori A.M., Caprio M., Strollo F., Moretti C., Frajese G., Isidori A., Fabbri A. (1999). Leptin and androgens in male obesity: Evidence for leptin contribution to reduced androgen levels. J. Clin. Endocrinol. Metab..

[55] Tena-Sempere M., Pinilla L., González L.C., Diéguez C., Casanueva F.F., Aguilar E. (1999). Leptin inhibits testosterone secretion from adult rat testis in vitro. J. Endocrinol..

[56] Ahima R.S. (2008). Revisiting leptin’s role in obesity and weight loss. J. Clin. Investig..

[57] Sánchez-Garrido M.A., Ruiz-Pino F., Manfredi-Lozano M., Leon S., Garcia-Galiano D., Castaño J.P., Luque R.M., Romero-Ruiz A., Castellano J.M., Diéguez C. (2014). Obesity-induced hypogonadism in the male: Premature reproductive neuroendocrine senescence and contribution of Kiss1-mediated mechanisms. Endocrinology.

[58] Behre H.M., Simoni M., Nieschlag E. (1997). Strong association between serum levels of leptin and testosterone in men. Clin. Endocrinol. (Oxf.).

[59] Hedger M.P., Meinhardt A. (2003). Cytokines and the immune-testicular axis. J. Reprod. Immunol..

[60] Guazzone V.A., Jacobo P., Theas M.S., Lustig L. (2009). Cytokines and chemokines in testicular inflammation: A brief review. Microsc. Res. Tech..

[61] Morelli A., Sarchielli E., Comeglio P., Filippi S., Vignozzi L., Marini M., Rastrelli G., Maneschi E., Cellai I., Persani L. (2014). Metabolic syndrome induces inflammation and impairs gonadotropin-releasing hormone neurons in the preoptic area of the hypothalamus in rabbits. Mol. Cell. Endocrinol..

[62] Watanobe H., Hayakawa Y. (2003). Hypothalamic interleukin-1 beta and tumor necrosis factor-alpha, but not interleukin-6, mediate the endotoxin-induced suppression of the reproductive axis in rats. Endocrinology.

[63] Russell S.H., Small C.J., Stanley S.A., Franks S., Ghatei M.A., Bloom S.R. (2001). The in vitro role of tumour necrosis factor-alpha and interleukin-6 in the hypothalamic-pituitary gonadal axis. J. Neuroendocrinol..

[64] Leisegang K., Henkel R. (2018). The in vitro modulation of steroidogenesis by inflammatory cytokines and insulin in TM3 Leydig cells. Reprod. Biol. Endocrinol..

[65] Fan W., Xu Y., Liu Y., Zhang Z., Lu L., Ding Z. (2018). Obesity or overweight, a chronic inflammatory status in male reproductive system, leads to mice and human subfertility. Front. Physiol..

[66] Syriou V., Papanikolaou D., Kozyraki A., Goulis D.G. (2018). Cytokines and male infertility. Eur. Cytokine Netw..

[67] Ebrahimi F., Urwyler S.A., Straumann S., Doerpfeld S., Bernasconi L., Neyer P., Schuetz P., Mueller B., Donath M.Y., Christ-Crain M. (2018). IL-1 antagonism in men with metabolic syndrome and low testosterone: A randomized clinical trial. J. Clin. Endocrinol. Metab..

[68] Navarro G., Xu W., Jacobson D.A., Wicksteed B., Allard C., Zhang G., De Gendt K., Kim S.H., Wu H., Zhang H. (2016). Extranuclear actions of the androgen receptor enhance glucose-stimulated insulin secretion in the male. Cell Metab..

[69] Lin H.Y., Yu I.C., Wang R.S., Chen Y.T., Liu N.C., Altuwaijri S., Hsu C.L., Ma W.L., Jokinen J., Sparks J.D. (2008). Increased hepatic steatosis and insulin resistance in mice lacking hepatic androgen receptor. Hepatology.

[70] MacLean H.E., Chiu W.S., Notini A.J., Axell A.M., Davey R.A., McManus J.F., Ma C., Plant D.R., Lynch G.S., Zajac J.D. (2008). Impaired skeletal muscle development and function in male, but not female, genomic androgen receptor knockout mice. FASEB J..

[71] Rodriguez-Cuenca S., Monjo M., Proenza A.M., Roca P. (2005). Depot differences in steroid receptor expression in adipose tissue: Possible role of the local steroid milieu. Am. J. Physiol. Endocrinol. Metab..

[72] Yu I.C., Lin H.Y., Liu N.C., Wang R.S., Sparks J.D., Yeh S., Chang C. (2008). Hyperleptinemia without obesity in male mice lacking androgen receptor in adipose tissue. Endocrinology.

[73] McInnes K.J., Smith L.B., Hunger N.I., Saunders P.T., Andrew R., Walker B.R. (2012). Deletion of the androgen receptor in adipose tissue in male mice elevates retinol binding protein 4 and reveals independent effects on visceral fat mass and on glucose homeostasis. Diabetes.

[74] Yu I.C., Lin H.Y., Liu N.C., Sparks J.D., Yeh S., Fang L.Y., Chen L., Chang C. (2013). Neuronal androgen receptor regulates insulin sensitivity via suppression of hypothalamic NF-κB-mediated PTP1B expression. Diabetes.

[75] Kelly D.M., Jones T.H. (2013). Testosterone: A metabolic hormone in health and disease. J. Endocrinol..

[76] Gyawali P., Martin S.A., Heilbronn L.K., Vincent A.D., Taylor A.W., Adams R.J.T., O’Loughlin P.D., Wittert G.A. (2018). The role of sex hormone-binding globulin (SHBG), testosterone, and other sex steroids, on the development of type 2 diabetes in a cohort of community-dwelling middle-aged to elderly men. Acta Diabetol..

[77] Hamilton E.J., Gianatti E., Strauss B.J., Wentworth J., Lim-Joon D., Bolton D., Zajac J.D., Grossmann M. (2011). Increase in visceral and subcutaneous abdominal fat in men with prostate cancer treated with androgen deprivation therapy. Clin. Endocrinol..

[78] Saylor P.J., Smith M.R. (2013). Metabolic complications of androgen deprivation therapy for prostate cancer. J. Urol..

[79] Hackett G. (2019). Type 2 diabetes and testosterone therapy. World J. Men’s Health.

[80] Traish A.M., Kypreos K.E. (2011). Testosterone and cardiovascular disease: An old idea with modern clinical implications. Atherosclerosis.

[81] Corona G., Rastrelli G., Monami M., Saad F., Luconi M., Lucchese M., Facchiano E., Sforza A., Forti G., Mannucci E. (2013). Body weight loss reverts obesity-associated hypogonadotropic hypogonadism: A systematic review and meta-analysis. Eur. J. Endocrinol..

[82] Mora M., Aranda G.B., de Hollanda A., Flores L., Puig-Domingo M., Vidal J. (2013). Weight loss is a major contributor to improved sexual function after bariatric surgery. Surg. Endosc..

[83] Stanik S., Dornfeld L.P., Maxwell M.H., Viosca S.P., Korenman S.G. (1981). The effect of weight loss on reproductive hormones in obese men. J. Clin. Endocrinol. Metab..

[84] Kaukua J., Pekkarinen T., Sane T., Mustajoki P. (2003). Sex hormones and sexual function in obese men losing weight. Obes. Res..

[85] Khoo J., Piantadosi C., Worthley S., Wittert G.A. (2010). Effects of a low energy diet on sexual function and lower urinary tract symptoms in obese men. Int. J. Obes..

[86] Khoo J., Piantadosi C., Duncan R., Worthley S.G., Jenkins A., Noakes M., Worthley M.I., Lange K., Wittert G.A. (2011). Comparing effects of a low-energy diet and a high-protein low-fat diet on sexual and endothelial function, urinary tract symptoms, and inflammation in obese diabetic men. J. Sex. Med..

[87] Niskanen L., Laaksonen D.E., Punnonen K., Mustajoki P., Kaukua J., Rissanen A. (2004). Changes in sex hormone-binding globulin and testosterone during weight loss and weight maintenance in abdominally obese men with the metabolic syndrome. Diabetes Obes. Metab..

[88] Corona G., Rastrelli G., Morelli A., Sarchielli E., Cipriani S., Vignozzi L., Maggi M. (2019). Treatment of functional hypogonadism besides pharmacological substitution. World J. Men’s Health.

[89] Howie B.J., Shultz T.D. (1985). Dietary and hormonal interrelationships among vegetarian Seventh-Day Adventists and nonvegetarian men. Am. J. Clin. Nutr..

[90] Bélanger A., Locong A., Noel C., Cusan L., Dupont A., Prévost J., Caron S., Sévigny J. (1989). Influence of diet on plasma steroids and sex hormone-binding globulin levels in adult men. J. Steroid. Biochem..

[91] Key T.J., Roe L., Thorogood M., Moore J.W., Clark G.M., Wang D.Y. (1990). Testosterone, sex hormone-binding globulin, calculated free testosterone, and oestradiol in male vegans and omnivores. Br. J. Nutr..

[92] Varlamov O. (2017). Western-style diet, sex steroids and metabolism. Biochim. Biophys. Acta Mol. Basis Dis..

[93] Kumagai H., Yoshikawa T., Zempo-Miyaki A., Myoenzono K., Tsujimoto T., Tanaka K., Maeda S. (2018). Vigorous physical activity is associated with regular aerobic exercise-induced increased serum testosterone levels in overweight/obese men. Horm. Metab. Res..

[94] Armamento-Villareal R., Aguirre L.E., Qualls C., Villareal D.T. (2016). Effect of lifestyle intervention on the hormonal profile of frail, obese older men. J. Nutr. Health Aging.

[95] Di Daniele N., Noce A., Vidiri M.F., Moriconi E., Marrone G., Annicchiarico-Petruzzelli M., D’Urso G., Tesauro M., Rovella V., De Lorenzo A. (2017). Impact of mediterranean diet on metabolic syndrome, cancer and longevity. Oncotarget.

[96] De Lorenzo A., Noce A., Moriconi E., Rampello T., Marrone G., Di Daniele N., Rovella V. (2018). MOSH syndrome (male obesity secondary hypogonadism): Clinical asssessment and possible therapeutic approaches. Nutrients.

[97] Kim C., Barrett-Connor E., Aroda V.R., Mather K.J., Christophi C.A., Horton E.S., Pi-Sunyer X., Bray G.A., Labrie F., Golden S.H. (2016). Diabetes Prevention Program Research Group. Testosterone and depressive symptoms among men in the Diabetes Prevention Program. Psychoneuroendocrinology.

[98] Hakonsen L.B., Thulstrup A.M., Aggerholm A.S., Olsen J., Bonde J.P., Andersen C.Y., Bungum M., Ernst E.H., Hansen M.L., Ernst E.H. (2011). Does weight loss improve semen quality and reproductive hormones? Results from a cohort of severely obese men. Reprod. Health.

[99] Hildreth K.L., Barry D.W., Moreau K.L., Vande Griend J., Meacham R.B., Nakamura T., Wolfe P., Kohrt W.M., Ruscin J.M., Kittelson J. (2013). Effects of testosterone and progressive resistance exercise in healthy, highly functioning older men with low-normal testosterone levels. J. Clin. Endocrinol. Metab..

[100] Hoyos C.M., Killick R., Yee B.J., Grunstein R.R., Liu P.Y. (2012). Effects of testosterone therapy on sleep and breathing in obese men with severe obstructive sleep apnoea: A randomized placebo-controlled trial. Clin. Endocrinol..

[101] Ng Tang Fui M., Hoermann R., Prendergast L.A., Zajac J.D., Grossmann M. (2017). Symptomatic response to testosterone treatment in dieting obese men with low testosterone levels in a randomized, placebo-controlled clinical trial. Int. J. Obes..

[102] Ng Tang Fui M., Hoermann R., Zajac J.D., Grossmann M. (2017). The effects of testosterone on body composition in obese men are not sustained after cessation of testosterone treatment. Clin. Endocrinol..

[103] Yassin A., Almehmadi Y., Saad F., Doros G., Gooren L. (2016). Effects of intermission and resumption of long-term testosterone replacement therapy on body weight and metabolic parameters in hypogonadal in middle-aged and elderly men. Clin. Endocrinol..

[104] Krasnoff J.B., Basaria S., Pencina M.J., Jasuja G.K., Vasan R.S., Ulloor J., Zhang A., Coviello A., Kelly-Hayes M., D’Agostino R.B. (2010). Free testosterone levels are associated with mobility limitation and physical performance in community-dwelling men: The Framingham Offspring Study. J. Clin. Endocrinol. Metab..

[105] Snyder P.J., Bhasin S., Cunningham G.R., Matsumoto A.M., Stephens-Shields A.J., Cauley J.A., Gill T.M., Barrett-Connor E., Swerdloff R.S., Wang C. (2016). Testosterone Trials Investigators. Effects of Testosterone Treatment in Older Men. N. Engl. J. Med..

[106] Nam Y.S., Lee G., Yun J.M., Cho B. (2018). Testosterone replacement, muscle strength, and physical function. World J. Men’s Health.

[107] Storer T.W., Basaria S., Traustadottir T., Harman S.M., Pencina K., Li Z., Travison T.G., Miciek R., Tsitouras P., Hally K. (2017). Effects of testosterone supplementation for 3 years on muscle performance and physical function in older men. J. Clin. Endocrinol. Metab..

[108] Haider A., Saad F., Doros G., Louis Gooren L. (2013). Hypogonadal obese men with and without diabetes mellitus type 2 lose weight and show improvement in cardiovascular risk factors when treated with testosterone: An observational study. Obes. Res. Clin. Pract..

[109] Dhindsa S., Miller M.G., McWhirter C.L., Mager D.E., Ghanim H., Chaudhuri A., Dandona P. (2010). Testosterone concentrations in diabetic and nondiabetic obese men. Diabetes Care.

[110] Kalinchenko S.Y., Tishova Y.A., Mskhalaya G.J., Gooren L.J., Giltay E.J., Saad F. (2010). Effects of testosterone supplementation on markers of the metabolic syndrome and inflammation in hypogonadal men with the metabolic syndrome: The double-blinded placebo-controlled Moscow study. Clin. Endocrinol..

[111] Aversa A., Bruzziches R., Francomano D., Rosano G., Isidori A.M., Lenzi A., Spera G. (2010). Effects of testosterone undecanoate on cardiovascular risk factors and atherosclerosis in middle-aged men with late-onset hypogonadism and metabolic syndrome: Results from a 24-month, randomized, double-blind, placebo-controlled study. J. Sex. Med..

[112] Haring R., Völzke H., Steveling A., Krebs A., Felix S.B., Schöfl C., Dörr M., Nauck M., Wallaschofski H. (2010). Low serum testosterone levels are associated with increased risk of mortality in a population-based cohort of men aged 20–79. Eur. Heart J..

[113] Laaksonen D.E., Niskanen L., Punnonen K., Nyyssönen K., Tuomainen T.P., Valkonen V.P., Salonen R., Salonen J.T. (2004). Testosterone and sex hormone-binding globulin predict the metabolic syndrome and diabetes in middle-aged men. Diabetes Care.

[114] Corona G., Rastrelli G., Monami M., Guay A., Buvat J., Sforza A., Forti G., Mannucci E., Maggi M. (2011). Hypogonadism as a risk factor for cardiovascular mortality in men: A meta-analytic study. Eur. J. Endocrinol..

[115] Mulligan T., Frick M.F., Zuraw Q.C., Stemhagen A., McWhirter C. (2006). Prevalence of hypogonadism in males aged at least 45 years: The HIM study. Int. J. Clin. Pract..

[116] Francomano D., Ilacqua A., Bruzziches R., Lenzi A., Aversa A. (2014). Effects of 5-year treatment with testosterone undecanoate on lower urinary tract symptoms in obese men with hypogonadism and metabolic syndrome. Urology.

[117] Corona G., Giagulli V.A., Maseroli E., Vignozzi L., Aversa A., Zitzmann M., Saad F., Mannucci E., Maggi M. (2016). Testosterone supplementation and body composition: Results from a meta-analysis study. Eur. J. Endocrinol..

[118] Yassin A., Haider A., Haider K.S., Caliber M., Doros G., Saad F., Garvey W.T. (2019). Testosterone therapy in men with hypogonadism prevents progression from prediabetes to type 2 diabetes: Eight-year data from a registry study. Diabetes Care.

[119] Corona G., Rastrelli G., Di Pasquale G., Sforza A., Mannucci E., Maggi M. (2018). Testosterone and cardiovascular risk: Meta-Analysis of interventional studies. J. Sex. Med..

[120] Cunningham G.R., Ellenberg S.S., Bhasin S., Matsumoto A.M., Parsons J.K., Preston P., Cauley J.A., Gill T.M., Swerdloff R.S., Wang C. (2019). Prostate-Specific Antigen levels during testosterone treatment of hypogonadal older men: Data from a controlled trial. J. Clin. Endocrinol. Metab..

[121] Bhasin S., Singh A.B., Mac R.P., Carter B., Lee M.I., Cunningham G.R. (2003). Managing the risks of prostate disease during testosterone replacement therapy in older men: Recommendations for a standardized monitoring plan. J. Androl..

[122] Jahn J.L., Giovannucci E.L., Stampfer M.J. (2015). The high prevalence of undiagnosed prostate cancer at autopsy: Implications for epidemiology and treatment of prostate cancer in the Prostate-specific Antigen-era. Int. J. Cancer.

[123] Fenton J.J., Weyrich M.S., Durbin S., Liu Y., Bang H., Melnikow J. (2018). Prostate-Specific Antigen-based screening for prostate cancer: Evidence report and systematic review for the US Preventive Services Task Force. JAMA.

[124] Grossman D.C., Curry S.J., Owens D.K., Bibbins-Domingo K., Caughey A.B., Davidson K.W., Doubeni C.A., Ebell M., Epling J.W., US Preventive Services Task Force (2018). Screening for prostate cancer: US Preventive Services Task Force Recommendation Statement. JAMA.

